# Comparison of treatment strategies for resectable locally advanced primary mucinous adenocarcinoma of the lung

**DOI:** 10.1002/cam4.5684

**Published:** 2023-02-15

**Authors:** Yu Gu, Hongcheng Zhu, Jiaying Deng, Junhua Zhang, Tianxiang Chen, Songtao Lai

**Affiliations:** ^1^ Department of Radiation Oncology Fudan University Shanghai Cancer Center Shanghai China; ^2^ Department of Oncology, Shanghai Medical College Fudan University Shanghai China; ^3^ Shanghai Clinical Research Center for Radiation Oncology Shanghai China; ^4^ Shanghai Key Laboratory of Radiation Oncology Shanghai China; ^5^ Shanghai Lung Cancer Center, Shanghai Chest Hospital, School of Medicine Shanghai Jiao Tong University Shanghai China

**Keywords:** chemotherapy, lung adenocarcinoma, mucinous adenocarcinoma, postoperative radiotherapy

## Abstract

**Background:**

Primary pure mucinous adenocarcinoma (PMA) is a rare type of lung cancer with unique clinical and prognostic features. Previous studies have shown that PMA have more early‐stage cancer compared with other adenocarcinoma (ADC) subtypes. The clinicopathological features and optimal treatment strategies of resectable locally advanced mucinous adenocarcinoma lack evidence and require further study.

**Methods:**

In this study, we collected information from patients with stage III‐N2 PMA who underwent radical surgery between 2004 and 2016 from the Surveillance, Epidemiology, and End Results (SEER) database. The clinicopathological parameters, treatments, overall survival (OS), and cancer‐specific survival (CSS) were evaluated.

**Results:**

Of 242,699 eligible lung adenocarcinoma patients, 124 with PMA and 3405 with other ADCs of stage III‐N2 received radical surgery were identified. Compared with other ADCs, PMA tended to appear more in the lower lobes, with higher degree of differentiation, less early T stage, and more positive lymph nodes numbers. Patients with PMA had significantly worse survival than other ADCs (OS = 45.0 vs. 57.1 months, *p* = 0.005, CSS = 51.8 vs. 65.5 months, *p* = 0.017). We explored the benefit population of postoperative radiotherapy (PORT) and found that the population with ≤7 positive lymph nodes could benefit from PORT, and OS was significantly improved (41.2 vs. 69.3 months, *p* = 0.034). For patients with >7 positive lymph nodes, PORT did not provide a survival benefit, while chemotherapy improved OS (10.9 vs. 23.3 months, *p* = 0.041). Multivariate analysis showed that race, tumor location, number of positive lymph nodes, and PORT were independent prognostic factors in patients with postoperative III‐N2 lung PMA.

**Conclusion:**

The prognosis of patients with resectable III‐N2 primary lung PMA was significantly worse than that of other ADCs, and PORT was an independent prognostic factor. Patients with ≤7 positive lymph nodes could benefit from PORT and those with >7 positive lymph nodes could benefit from chemotherapy.

## Graphical abstract

Stage III‐N2 lung pure mucinous adenocarcinoma (PMA) tended to appear more in lower lobes, with higher differentiation degree, earlier T stage, and more positive lymph nodes(LNs) than other adenocarcinoma. The prognosis of resectable III‐N2 PMAs was worse than other adenocarcinoma. Patients with ≤7 positive LNs benefited from PORT and those with >7 positive LNs benefited from chemotherapy.

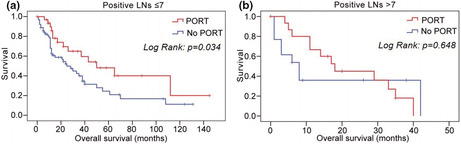

## 1 INTRODUCTION

With the development of industrialization and the increase in lung cancer screening, lung cancer is still cancer with the highest morbidity and mortality worldwide.^1^ Non‐small cell lung cancer (NSCLC) accounts for approximately 85% of lung cancers, among which the incidence of lung adenocarcinoma (ADCs) has increased significantly recently, becoming the most common histopathological type of NSCLC.^2^ Lung adenocarcinoma is a heterogeneous group of tumors with different subtypes of diverse molecular, clinical, and pathological characteristics. Primary pure mucinous adenocarcinoma (PMA) is an uncommon subtype of lung adenocarcinoma accounts for about 2%–10%.^3^ PMA refers to tumor cells with goblet or columnar cell morphology containing abundant intracytoplasmic and extracytoplasmic mucin, which has unique histological morphology and molecular phenotype. According to the latest report of the International Association for the Study of Lung Cancer/American Thoracic Society/European Respiratory Society (IASLC/ATS/ ERS) classification of lung adenocarcinoma, patients with PMA and other invasive adenocarcinoma have been separated for survival analysis.^4^, ^5^ Previous reports have suggested that the prognosis of PMA differs from that of other subtypes, and findings have been controversial.^4^, ^6^, ^7^, ^8^, ^9^ Some studies have reported that PMA have significantly better overall survival than nonmucinous adenocarcinoma,^4^, ^10^, ^11^, ^12^ while others have reported PMA is a more aggressive lung adenocarcinoma subtype with a worse prognosis.^6^, ^7^, ^13^, ^14^ Current research on PMA mainly focused on early‐stage cases. However, data on resectable local advanced PMA have not been reported and deserve further study.

There were many controversies about postoperative radiotherapy (PORT) in resectable local advanced especially stage III‐N2 NSCLC patients. More recently, LUNG‐ART and PORT‐C trials have shown that PORT does not provide survival benefits for resectable stage III‐N2 NSCLC.^15^, ^16^ However, the role of PORT in different adenocarcinoma subtypes needs to be further clarified. It has been reported that platinum‐based conventional chemotherapy has a poor effect and does not improve the overall survival of patients with stage IV PMA,^12^ while others have reported that chemotherapy is positively correlated with the overall survival rate of PMA.^10^ The role of chemotherapy in locally advanced resectable PMA also requires further study. In previous studies, PMA rarely have epidermal growth factor receptor(EGFR) mutations but frequently KRAS mutations(≥50%)^17^, ^18^, ^19^ which are quite different from nonmucinous adenocarcinomas. Therefore, the current recommended EGFR‐TKI adjuvant therapy might also be unsuitable for mucinous adenocarcinoma. Taken together, there is insufficient evidence for adjuvant therapy for locally advanced resectable lung mucinous adenocarcinoma, especially stage III‐N2 mucinous adenocarcinoma.

Given the relative low incidence, there are currently few large‐scale studies on PMA, and a large amount of data is required for further analysis. This study aimed to investigate treatment strategies for patients with resectable stage III‐N2 PMA of the lung from the Surveillance, Epidemiology, and End Results (SEER) database.

## 2 METHODS

### 2.1 Patients

The SEER * Stat 8.3.9 version was used to collect information from a representative patient population for this study (http://seer.cancer.gov/). SEER 18 Regs Custom Data (with additional treatment fields) were selected which covered nearly 28% of the population in the United States. Patients selected in this study were based on the inclusion criteria: (i) PMA were used to restrict the pathology types to (ICD‐O‐3 codes 8253/3, 8480/3), and other ADCs (ICD‐O‐3 codes 8140/3, 8250/3, 8251/3, 8252/3, 8255/3). (ii) American Joint Committee on Cancer (AJCC, 8th edition) stage III‐N2 (include cN2 and pN2). (iii) receiving a radical surgery of either a lobectomy or pneumonectomy between 2004 and 2016, surgery approach was defined according to surgery code; (iv) lung adenocarcinoma was only cancer or the first primary cancer. Exclusion criteria were as follows: (i) did not diagnose by histology; (ii) metastatic disease; (iii) received preoperative radiotherapy alone. Finally, a total of 124 PMA and 3405 other ADCs were included. The number of positive lymph nodes was analyzed by using the X‐tile plot^20^ to determine the appropriate cutoff value. Accordingly, PMA patients were stratified into the LNs ≤ 7 group and LNs > 7 group (Figure S1).

### 2.2 Statistical analysis

OS was defined as the time from the date of diagnosis to the date of death for any reason, and CSS was defined as the time from the date of diagnosis to the date of death for lung cancer. Kaplan–Meier methods were used to estimate survival, with log‐rank tests used for statistical comparisons. Cox proportional hazard model was conducted for univariate and multivariate analyses of patient survival‐related variables, and the corresponding results above were shown in the forest plot. A *p* value of <0.05 was regarded as statistically significant, and all statistical tests were two‐tailed. Statistical analysis was performed using IBM SPSS Statistics 21.0, and the Forest plots were drawn using R version 3.2.3.

## 3 RESULTS

### 3.1 Baseline characteristics

**FIGURE 1:**
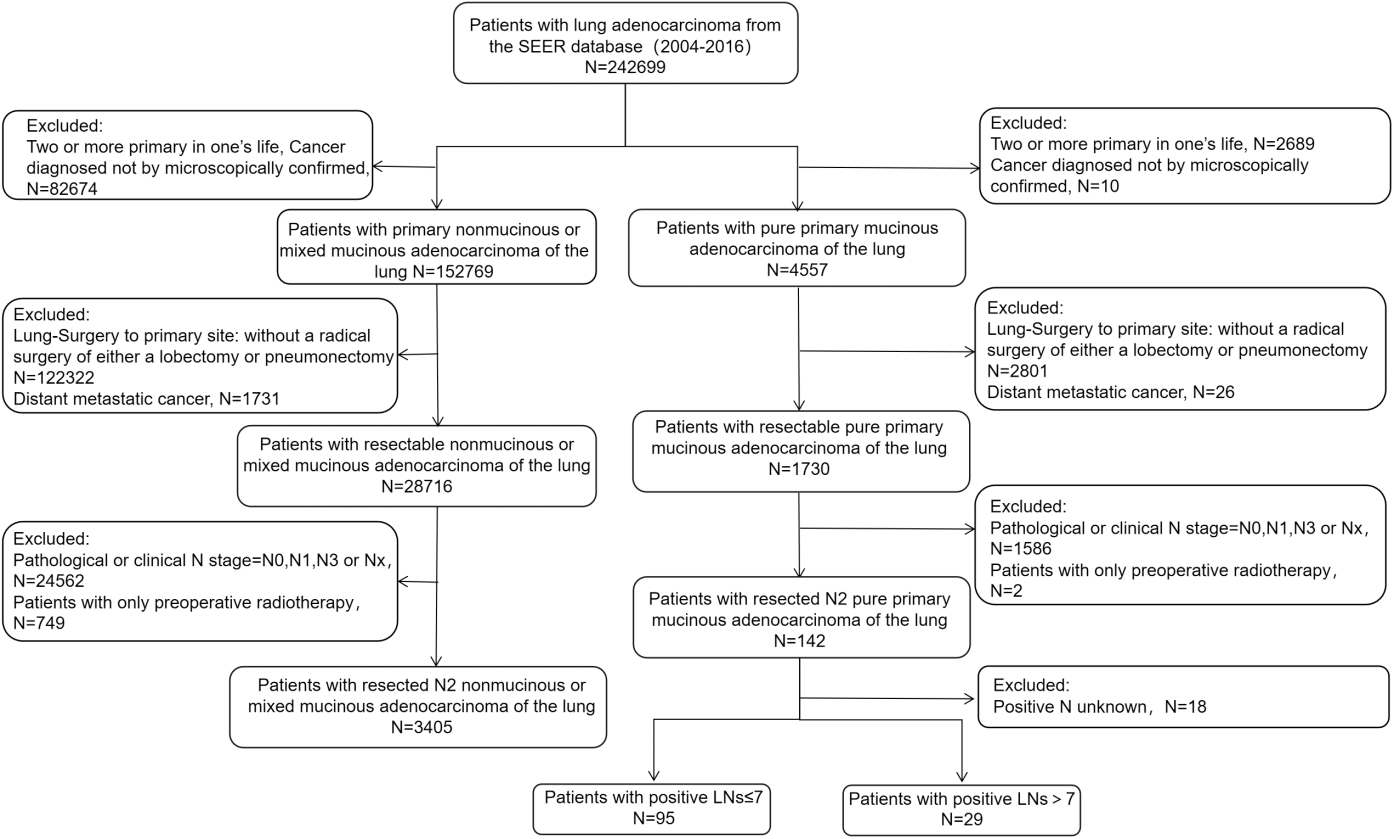
Flowchart of case inclusion and exclusion.

Overall, we identified 242,699 lung adenocarcinoma patients registered from 2004 to 2016. We restricted the patients with stage III‐N2, received a radical surgery of either lobectomy or pneumonectomy, and with one and only one malignancy. In addition, we excluded data from cases that received preoperative radiotherapy alone, and that did not provide the number of positive lymph nodes. According to the criteria, 124 PMA patients and 3405 other ADCs patients were included (Figure 1).

We compared the PMA group with other ADCs patients (*N* = 3405) at stage III‐N2 stage after radical resection, and statistically significant differences of clinicopathological features were summarized (Table 1). Patients with PMA and other ADCs were similar in age, sex, race, and tumor laterality (Table 1). Compared with other ADCs, PMA tented to be distributed in the lower lung lobe (54.8% vs. 32.6% with lower lobe, 36.3% vs. 58.4% with upper lobe, *p* < 0.001). In addition, the PMA group had more well‐differentiated pathology grade (17.7% vs.5.6% with well‐differentiated, 18.5% vs. 42.9% with poorly differentiated, *p* < 0.001), less early T stage (55.6% vs. 79.5% with T1 + T2, *p* < 0.001), and more positive lymph nodes (23.4% vs. 12.0% with positive lymph nodes>7, *p* < 0.001). In terms of treatment modalities, the proportions of patients in the two groups who received chemotherapy and PORT were similar.

**TABLE 1:** Comparison of clinicopathological characteristics between stage III‐N2 pure mucinous adenocarcinomas (PMA) and stage III‐N2 other adenocarcinomas (ADCs).

Characteristics	PMA (*N* = 124)	Other ADCs (*N* = 3405)	*p*
Age (years)			
<70	79 (63.7%)	2185 (64.2%)	0.916
≥70	45 (36.3%)	1220 (35.8%)	
Race			
Black	8 (6.5%)	304 (8.9%)	0.556
White	104 (83.9%)	2728 (80.1%)	
Other*	12 (9.7%)	367 (10.8%)	
Unknown	0 (0%)	6 (0.2%)	
Gender			
Female	63 (50.8%)	1949 (57.2%)	0.155
Male	61 (49.2%)	1456 (42.8%)	
Location			
Main bronchus	1 (0.8%)	14 (0.4%)	<0.001^*^
Upper lobe, lung	45 (36.3%)	1988 (58.4%)	
Middle lobe, lung	4 (3.2%)	170 (5.0%)	
Lower lobe, lung	68 (54.8%)	1111 (32.6%)	
Overlapping lesion	3 (2.4%)	74 (2.2%)	
Lung, NOS	3 (2.4%)	48 (1.4%)	
Histologic grade			
Well	22 (17.7%)	191 (5.6%)	<0.001^*^
Moderately	59 (47.6%)	1478 (43.4%)	
Poorly	23 (18.5%)	1460 (42.9%)	
Undifferentiated	3 (2.4%)	29 (0.9%)	
Unknown	17 (13.7%)	247 (7.3%)	
Laterality			
Left	55 (44.4%)	1461 (42.9%)	0.749
Right	69 (55.6%)	1944 (57.1%)	
T stage			
T1	33 (26.6%)	939 (27.6%)	<0.001^*^
T2	36 (29.0%)	1767 (51.9%)	
T3	21 (16.9%)	198 (5.8%)	
T4	32 (25.8%)	424 (12.5%)	
Tx	2 (1.6%)	17 (0.5%)	
Chemotherapy			
Performed	90 (72.6%)	2500 (73.4%)	0.835
No/unknown	34 (27.4%)	905 (26.6%)	
Radiotherapy			
PORT	48 (38.7%)	1319 (38.7%)	0.995
No	76 (61.3%)	2086 (61.3%)	
Positive LNs			
1–7	95 (76.6%)	2996 (88.0%)	<0.001^*^
>7	29 (23.4%)	409 (12.0%)	

* indicates *p* < 0.05.

### 3.2 Survival Analyses between PMA and other ADCs

**FIGURE 2:**
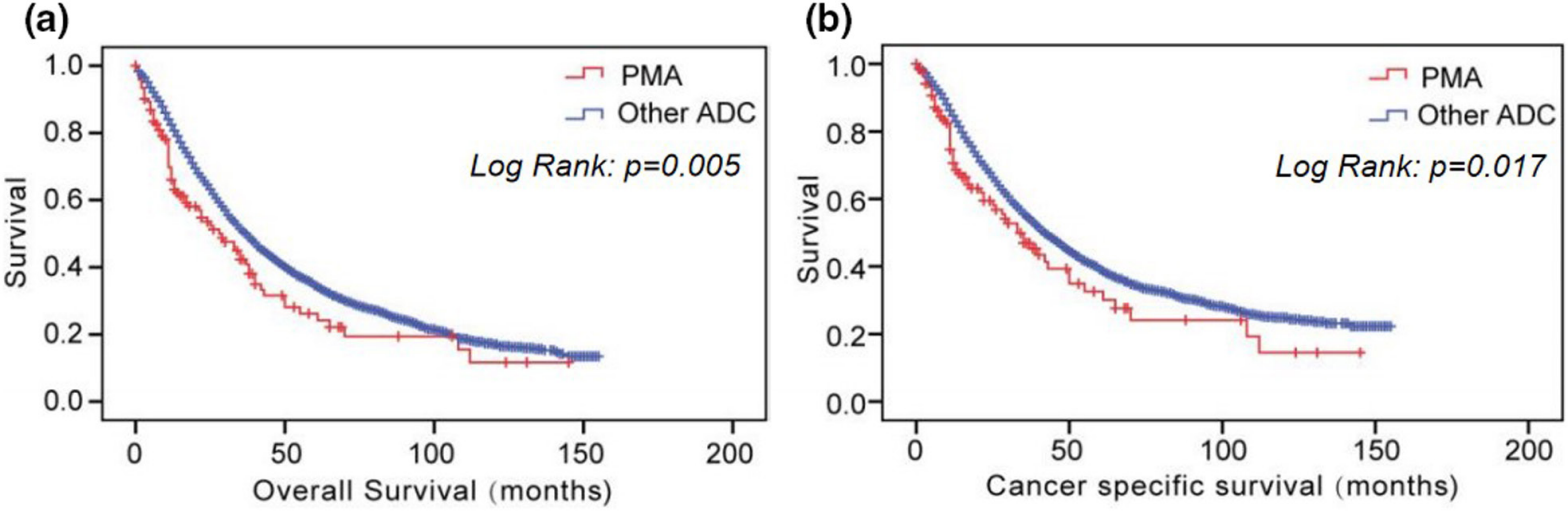
Overall survival of pure mucinous adenocarcinoma (PMA) patients and other adenocarcinoma (ADC) patients.

The survival analysis results of PMA patients and other ADC patients are shown in Figure 2. We found that in the resectable III‐N2 population, patients with PMA had significantly worse overall survival (OS) than patients with other ADCs, with a median OS of 45.0 months (95% CI: 34.4–55.6 months) for PMA and 57.1 months (95% CI: 55.0–59.1 months) for other ADCs (*p* = 0.005) (Figure 2A). Moreover, cancer‐specific survival of patients with PMA was also significantly worse than that of other ADCs patients (51.8 months (95% CI: 39.7–63.9 months) versus 65.5 months (95% CI: 63.1–67.8 months), *p* = 0.017) (Figure 2B). The 5‐year OS and CSS rates of PMA and other ADCs were 26.2% versus 34.6% and 32.6% versus 39.1%, respectively.

### 3.3 The effect of different treatments on the prognosis of PMA

For all III‐N2 PMA, overall survival was not statistically different between the PORT and no PORT subgroup despite a trend toward better survival in the PORT group (Figure S2).

**FIGURE 3:**
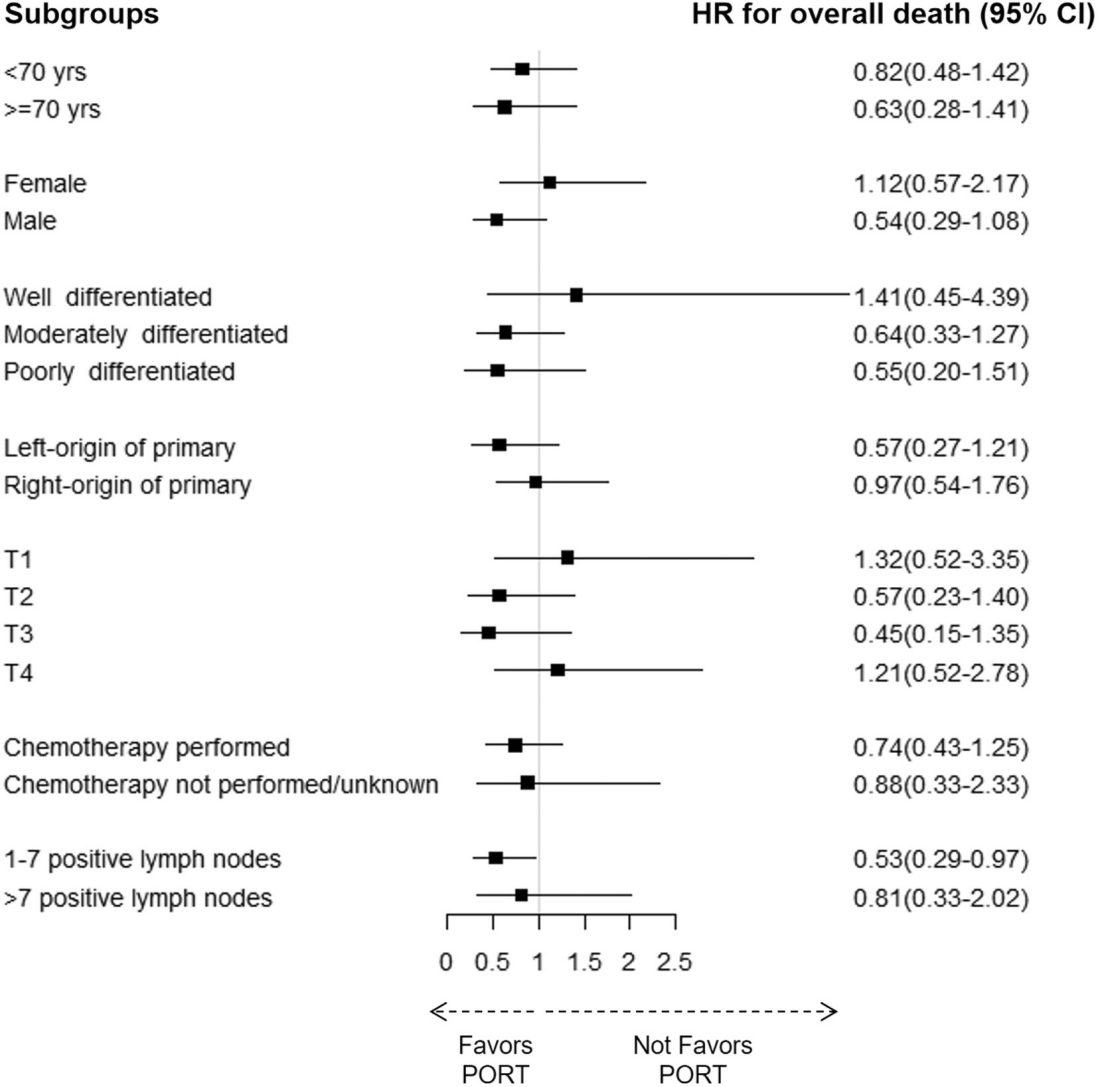
Result of subgroup analysis. Forest plots indicating the hazard ratio and 95% confidence interval of each subgroup. CI, confidence interval; HR, hazard ratio; PORT, postoperative radiotherapy.

To better understand the survival benefits of PORT for the PMA, we further analyzed the impact of PORT on survival stratified by age, sex, histologic grade, laterality, T stage, chemotherapy or not, and positive lymph nodes number. Patients with seven or less positive lymph nodes (LNs ≤ 7) were found to have an overall survival benefit from PORT (HR = 0.53, 95% CI: 0.29–0.97, *p* = 0.040) (Figure 3).

**FIGURE 4:**
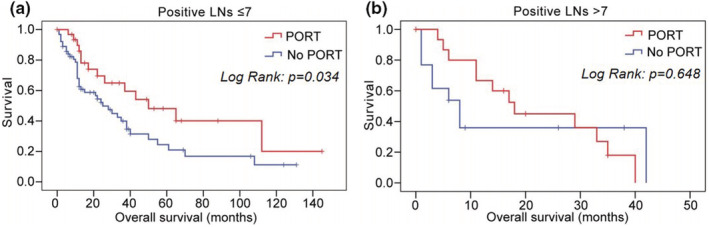
Overall survival of patients with and without PORT in different positive lymph nodes subgroups (A: ≤7, B: >7). LNs, lymph nodes; PORT, postoperative radiotherapy.

Patients with less positive lymph nodes (LN ≤ 7) showed significant improvement in overall survival after receiving PORT as 69.3 months (95% CI: 45.6–93.0 months), compared with no PORT as 41.2 months (95% CI: 28.5–53.8 months) (*p* = 0.034) (Figure 4). CSS has also improved in patients with positive LN ≤ 7 (49.7 vs. 75.3 months, no PORT vs. PORT, *p* = 0.078) (Figure S3, Table S1). Patients with more positive lymph nodes (LN > 7) had no survival benefit from PORT. In the patients with less than or equal to 7 positive lymph nodes, the 5‐year OS rates of no PORT group and PORT group were 24.5% versus 48.1%, respectively.

**FIGURE 5:**
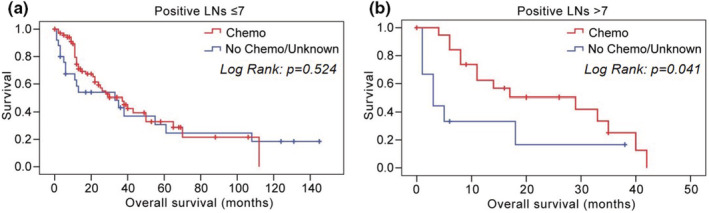
Overall survival of patients with and without chemotherapy in different positive lymph nodes subgroups (A: ≤7, B: >7). Chemo, chemotherapy; LNs, lymph nodes.

In addition, to explore the effect of chemotherapy on the prognosis of III‐N2 patients, we conducted the further analysis. We found that patients with more positive lymph nodes (LN > 7) had overall survival benefits from chemotherapy as 23.3 months (95% CI: 16.4–30.2 months), compared with no chemotherapy/unknown as 10.9 months (95% CI: 1.7–20.1 months) (*p* = 0.041) (Figure 5). CSS has also improved in patients with positive LN > 7 by chemotherapy (24.5 vs. 12.5 months, chemotherapy vs. no chemotherapy/unknown, *p* = 0.070) (Figure S4). Patients with less positive lymph nodes (LN ≤ 7) had no survival benefit from chemotherapy.

### 3.4 Identification of independent prognostic factors

Univariate analysis of prognostic factors for survival was conducted using the Cox proportional hazards model (Table 2). Multivariate analyses showed that race (*p* = 0.040), location (*p* = 0.045), positive lymph nodes (*p* = 0.003), and PORT (*p* = 0.031) were independent prognostic factors for overall survival, and location (*p* = 0.001), positive lymph nodes (*p* = 0.001), and PORT (*p* = 0.030) were independent prognostic factors for cancer‐specific survival. In sum, PORT was an independent prognostic factor for OS and CSS in III‐N2 PMA.

**TABLE 2:** Univariate and multivariate Cox proportional hazards analysis of overall survival (0S) and cancer‐specific survival (CSS) of PMA.

		OS				CSS			
		Univariate analysis	Multivariate analysis			Univariate analysis	Multivariate analysis		
Characteristics	Level	*p* value	HR	95% CI	*p* value	*p* value	HR	95% CI	*p* value
Age at diagnosis		0.436				0.591			
	<70 years								
	≥70 years								
Race		0.008			0.040^*^	0.032^*^			0.119
	Black		Reference				Reference		
	White		0.698	0.285–1.714	0.433		0.882	0.311–2.498	0.813
	Other		0.197	0.051–0.758	0.018^*^		0.298	0.072–1.233	0.095
Gender		0.513				0.681			
	Male								
	Female								
Location		0.003^*^			0.045^*^	<0.001^*^			0.001^*^
	Main bronchus		Reference				Reference		
	Upper lobe, lung		0.518	0.061–4.418	0.548		0.285	0.034–2.362	0.244
	Middle lobe, lung		3.365	0.337–33.544	0.301		3.904	0.384–39.665	0.250
	Lower lobe, lung		0.625	0.076–5.133	0.662		0.396	0.049–3.174	0.383
	Overlapping lesion		0.798	0.061–10.385	0.863		0.323	0.018–5.766	0.442
Histologic grade		0.274				0.162			
	Well								
	Moderately								
	Poorly								
	Undifferentiated								
Laterality		0.595				0.908			
	Left								
	Right								
T stage		0.100			0.468	0.437			
	T1		Reference						
	T2		0.955	0.492–1.856	0.893				
	T3		0.904	0.427–1.915	0.792				
	T4		1.484	0.763–2.885	0.245				
Positive LNs		0.004^*^			0.003^*^	0.001^*^			0.001^*^
	1–7		Reference				Reference		
	>7		2.380	1.336–4.240	0.003^*^		2.661	1.455–4.866	0.001^*^
PORT		0.107			0.031^*^	0.213			0.030^*^
	Not performed		Reference				Reference		
	Performed		0.558	0.328–0.948	0.031^*^		0.536	0.305–0.943	0.030^*^
Chemotherapy		0.221				0.657			
	Not performed/Unknown								
	Performed								

*indicates *p* < 0.05.

## 4 DISCUSSION

Lung cancer is the leading cause of cancer‐related death worldwide. PMA is a unique variant of adenocarcinoma with a low incidence, accounting for 2%–10% of lung adenocarcinomas.^13^, ^21^, ^22^ Lung mucinous adenocarcinoma was first reported by a case report in 1989.^23^ The definition of lung mucinous adenocarcinoma has been modified several times over the past few decades. The classification of lung cancer was updated by World Health Organization in 2015, with lung mucinous adenocarcinoma being divided into mucinous adenocarcinoma in situ, microinvasive mucinous adenocarcinoma, invasive mucinous adenocarcinoma, and colloidal adenocarcinoma.^24^ The histological features of PMA are mucin production by goblet cells and tall columnar epithelial cells.^18^, ^22^ Most of the previous studies described early‐stage resectable cases, and the characteristics of locally advanced resectable PMA were relatively unclear. To our knowledge, this is the first study to focus on treatment strategies for locally advanced resectable PMA.

Previous literature reported that lung mucinous adenocarcinoma mainly was located in the lower lung lobe on CT images, consistent with what we found in locally advanced resectable PMA. The possible reason might be that goblet cells are mainly distributed in the lower lobe, and the lower lobe is more susceptible to abnormal changes after being stimulated by foreign bodies due to gravity.^25^, ^26^ Tumors located in different lung lobes have different patterns of lymph node metastasis. Various studies have shown that lung cancers occurring in the different lobe are associated with a different prognosis.^27^, ^28^ Our study also found a correlation between tumor location and prognosis, but a larger sample size validation is needed for further analysis. In addition, we found that locally advanced resectable PMA tended to be more well‐differentiated than other ADCs, similar to previous reports in early‐stage PMA.^11^, ^12^, ^29^ Our study also showed that patients with PMA had a higher number of positive lymph nodes after surgery, which may be associated with their poor prognosis.

Given that PMA accounts for only 2%–10% of all lung adenocarcinoma cases, survival data for patients with PMA are limited and often conflicting. Chen et al. analyzed all stages of PMA found that the prognosis of PMA was better than that of other LUADs, but only 14% of them were stage III,^10^ and different stages of PMA prognosis might varies. However, other studies have shown that PMA has a similar or better prognosis than other subtypes of adenocarcinoma, but stage I‐II cases also account for the majority in these studies.^4^, ^8^, ^11^, ^30^ Other studies have shown that mucinous adenocarcinoma has a worse prognosis than other types of adenocarcinoma.^6^, ^7^, ^13^, ^14^ Yoshizawa et al. studied 514 cases of stage I lung cancer, including 13 cases of invasive mucinous adenocarcinoma. They classified invasive mucinous adenocarcinoma into a high‐grade group because of its high recurrence rate.^6^ Similarly, Russell et al. suggested that mucinous adenocarcinoma is a subtype with worse prognosis in lung adenocarcinoma.^7^ Our study shows that the prognosis of locally advanced resectable PMA is worse than that of other types of adenocarcinoma; however, the proportion of radiotherapy and chemotherapy involved of PMA in this study is the same as that of other adenocarcinomas, which indicates that the treatment strategy of PMA with advanced stage deserves further optimization.

Lung cancer patients still have a high rate of local recurrence and distant metastasis after surgery. Clinicians hope to use postoperative radiotherapy to increase the local control rate, reduce local recurrence, and further improve the survival of patients. Throughout the developmental history of radiotherapy, radiotherapy equipment and techniques have been continuously optimized and improved, and these changes have inevitably affected the results of some early and recent clinical studies. Therefore, we included data after 2004 to avoid bias due to backward radiotherapy techniques. The indications of PORT for III‐N2 non‐small cell lung cancer have been controversial. Multiple multicenter retrospective studies have evaluated the value of PORT in patients with III‐N2 NSCLC under the condition of three dimensional conformal RT (3D‐CRT)/intensity‐modulated radiotherapy (IMRT), and the results of these retrospective studies have shown that PORT may improve OS in patients with stage III‐N2 NSCLC.^31^, ^32^, ^33^ However, the recently published LUNG‐ART and PORT‐C trials showed that PORT did not improve postoperative recurrence and survival in patients with III‐N2 NSCLC. Nonetheless, the value of PORT in high‐risk subgroups continues to be explored. In the PORT‐C trial, a prospective exploratory analysis found that patients with more than or equal to 4 lymph node metastases had significantly improved disease‐free survival with PORT compared with patients with 1–3 lymph node metastases.^15^ Another study found that PORT in stage III‐N2 NSCLC improved DFS in patients with multisite N2 metastases compared with patients with single‐site N2 metastases (5‐year DFS 41% vs. 6%).^34^ Our study showed that the population with less positive lymph nodes (≤7) had a significant improvement in OS after receiving PORT, indicating that PORT has therapeutic value in III‐N2 PMA. However, the survival benefit of PORT was not significant in patients with more positive lymph nodes (LN > 7). Patients with a higher number of positive nodes are at increased risk of distant metastases, which may offset the benefit of local therapy.

There are also conflicting results on the role of chemotherapy in mucinous adenocarcinoma. It was reported that chemotherapy could improve the prognosis of patients with PMA, but this study included patients with stage I–IV.^10^ On the contrary, studies have shown that platinum‐containing chemotherapy did not improve overall survival in advanced mucinous adenocarcinoma compared with no chemotherapy.^12^ However, the confirmed biopsy specimens of advanced patients are small and may not reflect the complete picture of the tumor, resulting in diagnostic bias. At present, the role of chemotherapy in locally advanced resectable lung cancer is unclear. Our study shows that the population with more positive lymph nodes (LN > 7) can benefit from chemotherapy, which makes up for the deficiency of PORT in this population. These results suggest that systemic therapy is more important for this group of patients.

This study has several limitations. First, the SEER database did not provide detail clinical information of patients like smoking history, chemotherapy regimens, and timing of chemotherapy intervention, so we could not analyze these variables in the study. For example, our study found that blacks have a worse prognosis, which may be related to their genetic environment and need more information to explain. Second, this is a retrospective analysis study that was unbalanced in the number of patients in the two groups (PMA and other types of ADCs). It is unavoidable for a study focusing on rare subtypes to introduce bias into the statistical results. However, to our knowledge, this is the first report discussing treatment strategy for resectable locally advanced PMA. Although our study was neither prospective nor randomized, it was population‐based and reflected real‐world clinical characteristics, practices, and prognosis. This fact makes up for its limitations and allows us to establish a preliminary relationship between tumor pathological features and treatment strategies, and provides some evidence for treatment strategies in resectable locally advanced PMA. More research data validation is still needed in the future.

## 5 CONCLUSIONS

In conclusion, our population‐based study indicates patients with resectable III‐N2 lung PMA had significantly worse prognosis than that of other ADCs. For the same pathological stage group, PORT may provide survival benefit for patients with ≤7 positive lymph nodes, while chemotherapy may prove survival of those with >7 positive lymph nodes.

## AUTHOR CONTRIBUTIONS

**Yu Gu:** Conceptualization (equal); funding acquisition (equal); writing – original draft (lead). **Hongcheng Zhu:** Data curation (equal); writing – original draft (supporting). **Jiaying Deng:** Data curation (equal); formal analysis (equal). **Junhua Zhang:** Data curation (equal); formal analysis (equal). **Tianxiang Chen:** Conceptualization (equal); data curation (equal); funding acquisition (equal); validation (equal); writing – review and editing (equal). **Songtao Lai:** Conceptualization (equal); supervision (equal); writing – review and editing (equal).

## CONFLICT OF INTEREST STATEMENT

The authors declare no competing interests.

## Supporting information


Figure S1.

Figure S2.

Figure S3.

Figure S4.

Table S1.

## Data Availability

The datasets used during the present study are available from the corresponding author upon reasonable request.
