# Supplementary figures and images for: Comparison of treatment strategies for resectable locally advanced primary mucinous adenocarcinoma of the lung
Source: Cancer Med. 2023 Feb 15;12(8):9303–12. doi: 10.1002/cam4.5684 (PMC10166977; doi:10.1002/cam4.5684)

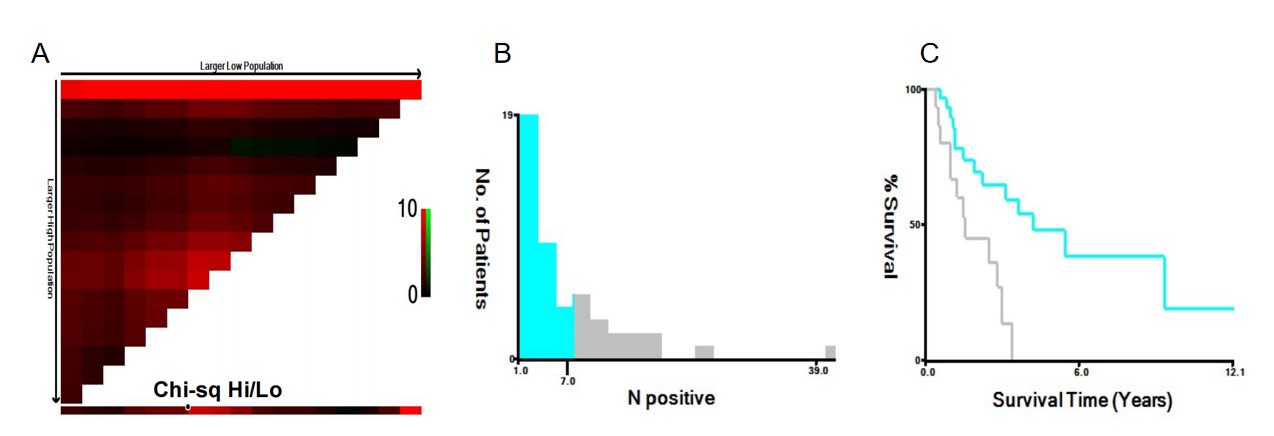

Supplement: Supplementary file 1 — Figure S1. [file CAM4-12-9303-s001.jpg]

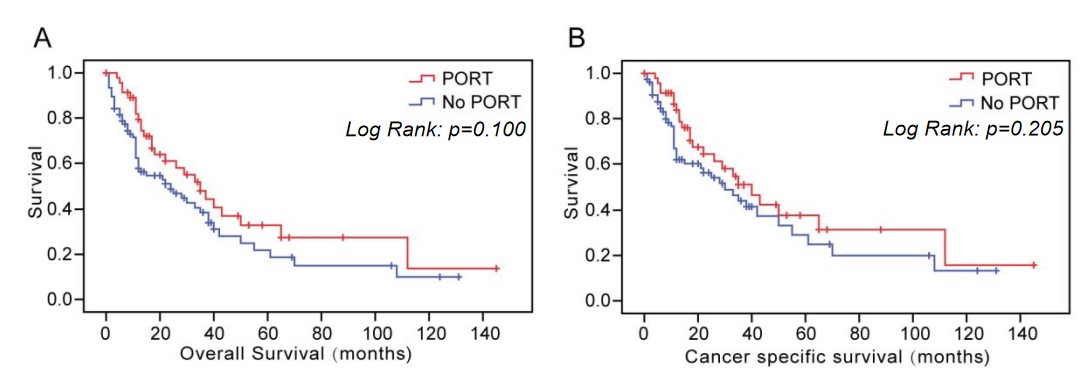

Supplement: Supplementary file 2 — Figure S2. [file CAM4-12-9303-s002.jpg]

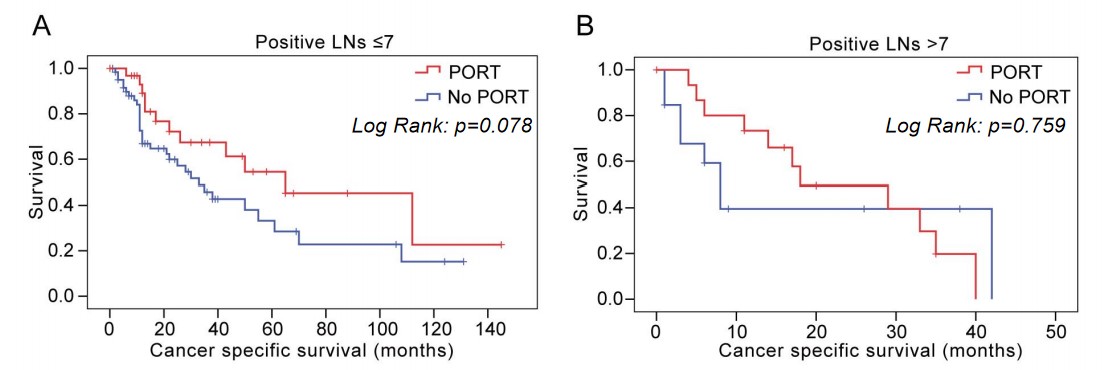

Supplement: Supplementary file 3 — Figure S3. [file CAM4-12-9303-s004.jpg]

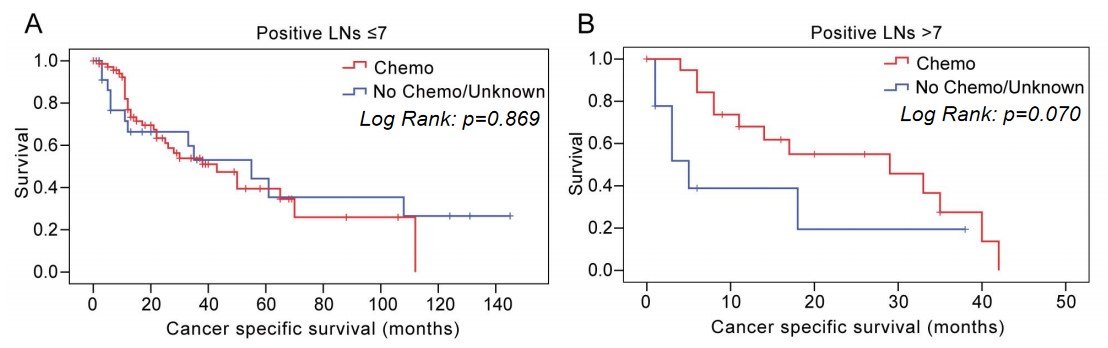

Supplement: Supplementary file 4 — Figure S4. [file CAM4-12-9303-s005.jpg]
