# Supplementary material for: Comparison of treatment strategies for resectable locally advanced primary mucinous adenocarcinoma of the lung
Source: Cancer Med. 2023 Feb 15;12(8):9303–12. doi: 10.1002/cam4.5684 (PMC10166977; doi:10.1002/cam4.5684)
Supplement: Supplementary file 5 — Table S1. [file CAM4-12-9303-s003.docx]

Supplementary Table1. Overall survival(OS) and Cancer specific survival(CSS) of patients with and without PORT by different positive lymph notes subgroups.

|  | OS(months) | | | CSS(months) | | |
| --- | --- | --- | --- | --- | --- | --- |
|  | PORT | No PORT | p | PORT | No PORT | p |
| 1-7 positive LNs | 69.31 | 41.18 | *0.034* | 75.32 | 49.65 | 0.078 |
| >7 positive LNs | 21.96 | 17.67 | 0.648 | 23.17 | 19.33 | 0.759 |
| Total | 54.08 | 38.04 | 0.100 | 58.98 | 45.34 | 0.205 |
